# REPRODUCIBILITY OF PHYSIOLOGICAL VARIABLES OF THE SIX-MINUTE WALK TEST IN HEALTHY STUDENTS

**DOI:** 10.1590/1984-0462/2021/39/2019326

**Published:** 2020-08-28

**Authors:** Patrícia Morgana Rentz Keil, Janaína Cristina Scalco, Renata Maba Gonçalves Wamosy, Camila Isabel Santos Schivinski

**Affiliations:** a Universidade do Estado de Santa Catarina, Florianópolis, SC, Brazil.

**Keywords:** Child, Reproducibility of results, Walking, Criança, Reprodutibilidade dos testes, Caminhada

## Abstract

**Objective:**

To verify the reproducibility of the six-minute walk test (6MWT) performance and its physiological variables in healthy students.

**Methods:**

This is as prospective cross-sectional study. The sample consisted of healthy students aged 6-12 years old from public and private schools in the region of Florianópolis City, Santa Catarina State, (Southern Brazil). The medical state was considered according to the health records and scores on the International Study of Asthma and Allergies in Childhood (ISAAC) and the spirometric values of forced expiratory volume in the first second and forced vital capacity above 80% of what was predicted. Two 6MWTs were conducted with a 30-minute interval between them, following the recommendations from the American Thoracic Society. Physiologic variables were recorded using the portable telemetric gas analyzer K4b2 (Cosmed^®^, Italy). For analysis, the dyspnea index, the perception of effort and performance variables identified in both 6MWT were considered. Data distribution was verified with the Shapiro-Wilk test and statistical analysis included paired t-test or Wilcoxon test, and intraclass correlation coefficient (ICC). The significance level adopted was 5%.

**Results:**

A total of 22 students with a mean age of 10.2±1.5 years participated in the study. The covered distance and the variation of oxygen consumption reproducibility between the two 6MWTs presented ICC=0.76 and ICC=0.86, respectively. There was also similar behavior of the physiological variables when comparing the two tests (p=0.001), especially the minute volume (MV), the oxygen consumption (VO_2_), and the carbon dioxide production (VCO2).

**Conclusions:**

The 6MWT showed reproducible values, both in performance and physiological parameters, in the healthy students analyzed.

## RESUMO

**Objetivo:**

Verificar a reprodutibilidade do desempenho e das variáveis fisiológicas do teste de caminhada de seis minutos (TC_6_) realizado por escolares saudáveis.

**Métodos:**

Estudo transversal prospectivo. A amostra foi composta de escolares saudáveis, entre 6 e 12 anos, provenientes de escolas públicas e privadas da Grande Florianópolis, SC, Brasil. A higidez foi controlada por meio do recordatório de saúde, do questionário International Study of Asthma and Allergies in Childhood (ISAAC) e dos valores espirométricos de volume expiratório forçado no primeiro segundo (VEF_1_) e capacidade vital forçada (CVF) acima de 80% do predito. Foram registradas as variáveis fisiológicas utilizando-se o analisador de gases telemétrico portátil K4b2 (Cosmed^®^, Itália), e, para análise, consideraram-se o índice de dispneia, a percepção de esforço e as variáveis de desempenho identificadas nos dois TC_6_. Verificou-se a distribuição dos dados pelo teste de Shapiro-Wilk, e a análise estatística incluiu: teste *t* de Student pareado, ou teste de Wilcoxon, e o coeficiente de correlação intraclasse (ICC). O nível de significância adotado foi de 5%.

**Resultados:**

Participaram 22 escolares com idade média de 10,2±1,5 anos. Identificou-se reprodutibilidade da distância percorrida e da variação do consumo de oxigênio entre os dois TC_6_, com ICC=0,76 e ICC=0,87, respectivamente. Houve similaridade no comportamento das variáveis fisiológicas na comparação entre os dois testes (p=0,001), destacando-se o volume minuto (VE), o consumo de oxigênio (VO_2_) e a produção de dióxido de carbono (VCO_2_).

**Conclusões:**

O TC_6_ apresentou valores reprodutíveis tanto no desempenho como nos parâmetros fisiológicos nos escolares saudáveis estudados.

## INTRODUCTION

Functional status is a multidimensional concept that refers to how an individual is able to complete activities of daily living (ADL), which are essential to meet their physical, psychological and social needs.^1^ This term can be measured in four distinct segments: functional performance, functional reserve, functional utilization capacity and functional capacity, the latter concerning the individual’s maximum potential to perform ADL.^1^ ^,^ ^2^ Functional capacity can be assessed with field tests,^3^ like the Sit to Stand Test, the AVD-Glittre adapted for children, the Stepping Test and the Six-Minute Walk Test (6MWT).^4^ ^,^ ^5^ ^,^ ^6^ ^,^ ^7^

Considering that the 6MWT is easy to apply, is safe, has a low cost^8^ and measurement properties and reference equations established for different pediatric populations,^9^ it is the most used test in the assessment of the functional capacity of children and adolescents. The test performance is attributed to the distance covered (DC) in meters, during the six minutes, which is considered a sensitive and important measure to monitor responses to therapeutic interventions of children with different dysfunctions,^10^ ^,^ ^11^ in addition to being a predictive measure of morbidity and mortality.^12^

However, the 6MWT does not yet have protocols or specific guidelines for its application in Pediatrics, because the American Thoracic Society (ATS) and European Respiratory Society (2014) documents standardize the application of this test based on studies including adults with chronic respiratory disease.^10^ Today, it is recommended to perform two 6MWTs, with an interval of 30 minutes between them, considering a possible learning effect observed in the adult population.^13^ However, in Pediatrics, such behavior is still controversial.^8^ ^,^ ^14^ ^,^ ^15^ ^,^ ^16^ In this group, the influence of anthropometric factors on test performance is discussed, as they are growing and developing, and there is a need for more specific technical standards for the age group, such as verbal command and pictorial effort scales.^17^

That said, it is necessary to know the behavior of the cardiovascular, ventilatory and metabolic demands induced by the 6MWT when it is performed by the pediatric population, in addition to the real need for two tests on the same day, regarding their usual indication for evaluation and clinical monitoring of children with chronic diseases.^7^ ^,^ ^14^

The objective of the present study was to verify the reproducibility of performance and physiological variables (cardiovascular, ventilatory and metabolic) of the 6MWT performed by healthy students.

## METHOD

A prospective cross-sectional study was carried out for three months, which included healthy children, between 6 and 12 years old, from schools of the region of Florianópolis City, Santa Catarina State, Brazil, after approval by the Research Ethics Committee from Universidade do Estado de Santa Catarina, under Opinion No. 708.446 (Certificate of Presentation for Ethical Appreciation 22676113.6.0000.0118). Data collection took place by signing the informed consent form by parents and/or guardians and the child’s agreement to the consent form for minors. The participants’ healthiness was controlled with the application and analysis of:

- Health record, formulated by the researchers and considering the history and state of the individual with no illness.
- The International Study of Asthma and Allergies in Childhood (ISAAC) questionnaire - module I for asthma control - requiring a score less than 5 for children aged 6 to 9 and less than 6 points for adolescents aged 10 to 14.
- Spirometry exam, performed using the Easy One Frontline portable equipment (Medical Technologies^®^, Inc., United States), respecting the ATS recommendations.^18^ Those students who presented forced expiratory volume in the first second (FEV1) and forced vital capacity (FVC) with values above 80% of what was predicted were eligible.^19^ ^,^ ^20^

Based on data on healthiness control, students with percentiles≥3 and <97, classified as eutrophic and overweight, non-asthmatic and non-performing athletes (enrolled in sports federations) were included from the study. Children and adolescents with disabilities to perform any of the evaluation procedures would be excluded from the sample, which did not happen.

The participants’ anthropometric data, body mass and height were evaluated, followed by the calculation of the body mass index (BMI), with the Ministry of Health’s Telehealth program (http://www.telessaudebrasil.org.br/apps/calculadoras/). After that, two 6MWTs (6MWT1 and 6MWT2) were conducted, with an interval of 30 minutes between them, in the morning, according to the ATS recommendations. To perform the test, the student was instructed to go as far as possible during the six-minute period and encouraged to do so with standardized phrases said at every minute.^10^ All tests were conducted by the same previously trained evaluators, who measured the blood pressure (BP) and the sensation of dyspnea with the RPE (Rated Perceived Exertion) scale and the modified Borg scale - the first is scored from 0 to 5, and the second, from 0 to 10, with 10 representing the maximum symptom.^21^ ^,^ ^22^ The longest distance covered (DC) between the two tests was considered for analysis, which was recorded in meters.

For the analysis of physiological responses during the 6MWT, individuals used a K4b2 portable telemetric gas analyzer (Cosmed^®^, Italy). The variables respiratory rate (RR), heart rate (HR), minute volume (MV), oxygen consumption (VO_2_), VO_2_ in relation to body mass (VO_2_/kg), carbon dioxide production (VCO_2_), gas exchange rate (R), inspiratory time (iT), expiratory time (eT), total respiratory cycle time (totT), inspiratory time/total cycle time (iTi/totT) ratio, oxygen saturation (SpO2) and the metabolic equivalent (MET) were collected. For data analysis, variables were measured using the breath-to-breath technique, before and during the two 6MWTs, taking into account the average of the final 15 seconds of the initial rest and the average of the final 15 seconds of each minute of the 6MWT, collected using the gas analyzer.^23^

The sample calculation was based on an expected intraclass correlation coefficient (ICC) of 0.70 for DC in the 6MWT and for physiological variables, considering α=0.05 and β=0.10, totaling a sample of 17 students as sufficient for research.^24^

Statistical analysis was conducted using the IBM Statistical Package for the Social Sciences software (SPSS^®^, Chicago, IL, United States), version 20.0. Initially, data distribution was verified by the Shapiro-Wilk test, and, to compare the physiological variables between the beginning and the end of each of the two 6MWTs, the paired Student’s t test, or the Wilcoxon’s. The reproducibility of the 6MWT was analyzed with the ICC and the graphic layout of Bland-Altman. The ICC values obtained were interpreted according to the classification by Munro et al.,^25^ with little correlation=≤0.25, low=0.26-0.49, moderate=0.50-0.69, high=0.7-0.89 and very high=0.9-1.0. The level of significance adopted was 5%.

## RESULTS

A total of 22 healthy students with a mean age of 10.1 ± 1.4 years participated in this study. The average BMI of the students was 17.6 kg/m^2^ (± 2.20), with most participants classified as eutrophic (73.9%), and 26% of them, as overweight. The characteristics of the sample in relation to age, anthropometric variables and spirometric parameters are described in Table 1.

**Table 1:** Distribution of age data, anthropometric variables and spirometric parameters of the studied sample.

Parameters	Mean±SD	Median	(Minimum-Maximum)
Age (years old)	10.1±1.4	9.9	(7.5-12.9)
Body mass (kg)	35.2±9.0	32.7	(24.5-59.6)
Height (cm)	1.4±0.1	1.3	(1.2-1.6)
BMI (kg/cm^2^)	17.6±2.2	17.3	(14.1-22.3)
FEV_1_ (%pred)	96.3±9.2	98.0	(81-115)
FVC (%pred)	100.6±10.0	103.4	(83-118)

SD: standard deviation; kg: kilogram; cm: centimeters; BMI: body mass index; FEV_1_: forced expiratory volume in 1 second; FVC: forced vital capacity; %pred: percentage of what was predicted.^20^ ^,^ ^21^

### Reproducibility assessment between the 6MWT1 and the 6MWT2

**Figure 1:**
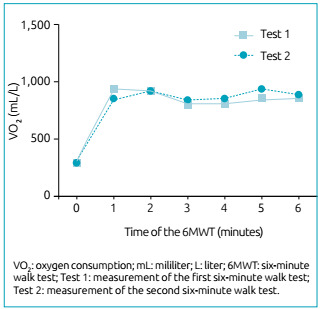
Representation of the behavior of the oxygen consumption variable between the two 6MWT.

**Figure 2:**
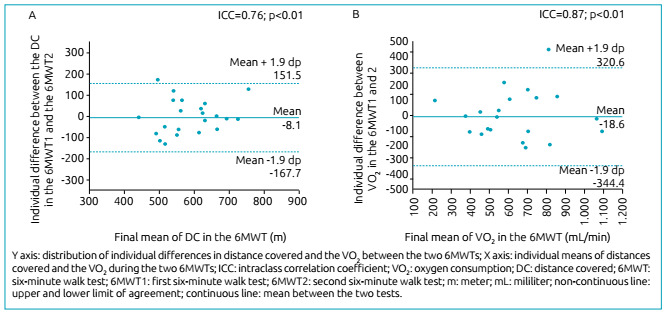
Bland-Altman (A) of the distance covered, and (B) of the behavior of the VO2 physiological parameter between the 6MWT1 and the 6MWT2.

By analyzing the reproducibility of DC and the variation of VO_2_ between the first and the second tests, a high reliability was identified between them, with ICC=0.76 (95% confidence interval - 95%CI, 0.41-0.90 m) and ICC=0.87 (95%CI, 0.68-0.94), respectively. The representation of VO_2_ behavior is shown in Figure 1, and its reproducibility and that of DC are displayed in Figure 2 by means of Bland-Altman graphs. Moderate to high reliability was also observed between the two 6MWTs, in most physiological parameters, as shown in Table 2, according to the ICC and the limits of agreement.

**Table 2:** Reproducibility of the performance and variation of physiological parameters between the 6MWT1 and the 6MWT2.

	6MWT1-6MWT2		
	ICC	95%CI	p-value
Performance (m)	0.76	0.41-0.90	0.001
ΔBORG	0.79	0.52-0.91	<0.001
ΔRPE scale	0.63	0.09-0.85	0.015
ΔRR (bpm)	0.62	0.12-0.84	0.012
ΔHR (bpm)	0.71	0.29-0.88	0.004
ΔMV (L/min)	0.83	0.61-0.93	<0.001
ΔVO_2_ (mL/min)	0.87	0.68-0.94	<0.001
ΔVO_2_/kg (mL/min/kg)	0.77	0.45-0.90	0.001
ΔVCO_2_ (mL/min)	0.84	0.62-0.93	<0.001
ΔR	0.72	0.34-0.88	0.003
ΔiT (s)	0.74	0.38-0.89	0.002
ΔeT (s)	0.47	-0.26-0.78	0.076
ΔtotT (s)	0.56	-0.05-0.81	0.034
ΔiT/totT (s)	0.66	0.21-0.86	0.007
ΔSpO_2_ (%)	0.23	-0.50-0.65	0.229
ΔMETS	0.77	0.45-0.90	0.001

6MWT1: first six-minute walk test; 6MWT2: second six-minute walk test; ICC: intraclass correlation coefficient; 95%CI: 95% confidence interval; p-*value*: reliability test value; Δ: variation (Δ=final-initial); BORG: dyspnea scale (points); RPE scale: rated perceived exertion scale (points); RR: respiratory rate; HR: heart rate; MV: minute volume; VO_2_: oxygen consumption; VO_2_/kg: VO_2_ in relation to body mass; VCO_2_: carbon dioxide production; R: gas exchange rate; iT: inspiratory time; eT: expiratory time; totT: total time of respiratory cicle; iT/totT: inspiratory time/total cycle time ratio; SpO_2_: oxygen saturation; METS: metabolic equivalent for oxygen; m: meters; bpm: breaths per minute; bpm: beats per minute; L: liters; min: minute; mL: mililiter; kg: kilogram; s: seconds.

There was no difference in performance between the two 6MWTs. The distances covered in the first (584.8 ± 85.1) and in the second test (584.9 ± 97.5) were similar. The analysis of the variation in cardiovascular, ventilatory and metabolic parameters between the 6MWT1 and the 6MWT2 did not identify any difference in any of the studied variables either. Table 3 contains the result of the comparison of the performance measures and the variation of the physiological parameters between the 6MWT1 and the 6MWT2, as well as the average data and the standard deviation.

**Table 3:** Result of the comparison of the performance measures and the variation of physiological parameters, between the 6MWT1 and the 6MWT2, as well as the data of mean and standard deviation.

	6MWT1		6MWT2		p-value
	Mean±SD	Min-Max	Mean±SD	Min-Max	
Performance	584.8±85.1	408.0-730.6	584±97.5	437-818	0.96
ΔBORG*	0.4±1.0	0.0-4.0	0.1±0.6	0.0-3.0	0.12
ΔRPE scale*	0.8±1.0	0.0-4.0	0.8±1.1	0.0-4.0	1.00
ΔRR	18.6±8.4	6.0-37.5	21.4±7.7	5.4-38.0	0.12
ΔHR	42.8±19.8	0.7-77.1	43.2±17.3	17.3-80.5	0.91
ΔMV	16.0±5.0	4.6-24.3	16.6±6.8	6.0-30.6	0.56
ΔVO_2_	643.3±232.5	163.3-1.138.9	656.0±260.9	261.4-1219.5	0.72
ΔVO_2_/kg	18.2±5.0	6.0-27.7	18.5±5.9	9.6-31.3	0.76
ΔVCO_2_	600.5±203.7	154.6-999.4	610.6±251.6	194.3-1.181.7	0.78
ΔR	-0.0±0.1	-0.3-0.1	-0.0±0.1	-0.2-0.1	0.71
ΔiT*	-0.4±0.3	-1.4-0.0	-0.4±0.3	-1.4-(-0.0)	0.68
ΔeT*	-0.9±0.5	-2.1-(-0.1)	-1.1±0.8	-4.2-(-0.3)	0.42
ΔtotT*	-1.4±0.8	-3.5-(-0.1)	-1.6±0.9	-5.0-(-0.3)	0.57
ΔiT/totT*	0.0±0.0	-0.0-0.1	0.0±0.0	0.0-0.2	0.45
ΔSpO_2_*	-0.5±1.1	-3.0-1.5	-1.6±1.8	-7.0-0.8	0.03
ΔMETS	5.2±1.4	1.7-7.9	5.3±1.6	2.7-8.9	0.76

6MWT1: first six-minute walk test; 6MWT2: second six-minute walk test; SD: standard deviation; Min: minimum; Max: maximum; p-*value*: statistical test value; * variables with non-parametric distribution analyzed with the Wilcoxon test; Δ: variation (Δ=final-initial); BORG: dyspnea scale (points); RPE scale: rated perceived exertion scale (points); RR: respiratory rate; HR: heart rate; MV: minute volume; VO_2_: oxygen consumption; VO_2_/kg: VO2 in relation to body mass; VCO_2_: carbon dioxide production; R: gas exchange rate; iT: inspiratory time; eT: expiratory time; totT: total time of respiratory cicle; iT/totT: inspiratory time/total cycle time ratio; SpO2: oxygen saturation; METS: metabolic equivalent for oxygen.

## DISCUSSION

The present study analyzed the reproducibility of DC and the physiological responses triggered by two 6MWTs performed by healthy children and adolescents. Based on the identification of low magnitude correlations in the eT and SpO2 variables, moderate in the RPE scale, RR, totT and iT/totT variables, as well as the high reliability in DC, Borg, HR, MV, VO_2_, VO_2_/Kg, VCO_2_, R, iT and metabolic equivalent for oxygen (METS), between the two tests performed by the same population of students, it can be said that the 6MWT is reproducible in this group. In the same vein, some studies had already found the reproducibility of this test in the healthy pediatric population,^15^ ^,^ ^16^ as well as in the behavior of cardiorespiratory parameters, assessed in a standard way. A study that included the presence of a gas analyzer to assess the behavior of the physiological parameters of healthy students during the 6MWT had not yet been conducted, which justifies this investigation and attributes it to being a pioneer.

Leunkeu et al.,^11^ among their analyzes, demonstrated the reproducibility of the DC variables and the physiological responses collected in the 6MWT, using a portable gas analyzer, in a sample of children and adolescents (14.2±1.8 years ) with cerebral palsy, classified in levels I and II of the Gross Motor Function Classification System (GMFCS). The values obtained in the two 6MWT were reproducible, with high reliability for DC (ICC=0.80 and DC=395±95 *vs*. 421±100 m, p=0.53), and also for the physiological response variables (VO_2_ peak: ICC=0.85; MV. peak: ICC=0.83; maximum HR: ICC=0.82). These results were like those obtained in the present study.

Among the physiological variables, oxygen consumption is interpreted in the literature as the main index of aerobic fitness during exercise.^26^ In view of this, in Pediatrics, the study by Bos et al.^27^ used VO_2_ peak to determine the level of physical activity and the aerobic fitness of children aged 6 to 12 undergoing liver transplantation. The assessment consisted of the gas analysis measured using the cardiopulmonary exercise test (CPET), considered the gold standard for this assessment. Among the variables, VO_2_, VO_2_ peak, MV and VCO_2_ were calculated, which defined that children have normal levels of aerobic conditioning.

The study of the measures obtained by gas analysis during tests to evaluate functional capacity and exercise in children enables the knowledge of the changes that these tests trigger in the arterial and venous partial pressures of gases, as well as possible ventilatory limitations during exertion (cardiovascular, respiratory and muscular or metabolic), in addition to assessing the behavior of stroke volume, obtained by analyzing the curves and the maximum values of the oxygen pulse (VO_2_/HR) and ventilatory equivalents (MV/VO_2_ and MV/VCO_2_), when an incremental protocol was performed.^28^ Therefore, the absence of CPET can be considered a limitation of the present study, considering that the data used as parameters for comparison with the responses obtained in the 6MWT were with prediction equations.

On the other hand, Pereira et al.^14^ assessed functional performance on the 6MWT in two different populations: students with cystic fibrosis (n=55) and healthy students (n=185), whose mean ages were 12.2±4.3 and 11.3±4.3 years, respectively. The results showed that the DC achieved between the first and the second tests were similar in both groups. These findings confirmed the reproducibility of the 6MWT, with high correlations, both for the group with cystic fibrosis (ICC=0.81) and the healthy one (ICC=0.77), and the ICC result for the healthy population was similar to that observed in the current investigation.

Along the same line, Cunha et al.^29^ evaluated the performance related to the clinical variables of children with cystic fibrosis (11.0±1.9 years), with a mean %FEV_1_of 63.1 (±21.1), and did not identified a significant difference between the means of DC (582.3±60 and 598.2±56.8 m), of cardiorespiratory responses and the sensation of dyspnea between the two 6MWTs. In obese students, the same pattern was verified by Morinder et al.^30^ in a study whose sample included ages between 8 and 16, and the test-retest was reproducible with high reliability (ICC=0.84) in this group. As to obesity, it is worth mentioning that almost 30% of the current research sample was characterized by being overweight, which can be considered a limitation, because it is known that this profile of individuals can present impairment in the performance of physical exercise.

The behavior of adult individuals during the 6MWT is well established, with a consensus on the presence of the learning effect in the performance of the test,^13^ which reinforces the guidelines regarding the need to perform two tests.^10^ In children with chronic kidney disease, this pattern was verified by Watanabe et al.,^8^ in a study that evaluated the reproducibility of the 6MWT in 38 children and adolescents (6 and 16 years old), who were on dialysis or had undergone kidney transplantation. The authors found that this population had a greater distance covered in the second test (519m (362-674) *vs*. 538.5m (405-685); p<0.001), with low reliability between them (ICC>0.4) . According to the authors, in this specific condition, there is a need for test-retest, as described by the ATS.^10^

In Pediatrics, the need for two tests is still discussed. In this sense, two studies evaluated healthy children and noted high reproducibility of DC in the 6MWT^15^ ^,^ ^16^ (ICC=0.82 and ICC=0.84, respectively), but did not show a learning effect in the referred investigations, with similar DC between the test-retest. Martins et al.^15^ attributed this behavior to the fact that healthy children are motivated by the novelty in view of the first test and do not commit themselves so much to the repetition of a second 6MWT, because the test is now known.

The results of the present study reflect the characteristics of a sample of healthy children, which can be considered a limitation of this investigation. Therefore, it is recommended to carry out further research in this line, including populations of chronic pulmonary patients and other specific situations, given the importance of monitoring the functional capacity in these individuals and identifying results similar to those presented here. This is because the application of a single test can be discussed for these individuals, which simplifies its application and performance, since the repetition of the 6MWT requires greater energy expenditure, which may not be indicated in critically ill patients. In addition, saving time spent for two 6MWTs increases its feasibility and applicability both in outpatient settings and in epidemiological studies conducted externally and, therefore, justifies further investigations.

The results shown here, which verified similarity in performance and in the response of physiological variables in the execution of two 6MWTs, suggest the possibility of conducting a single test in populations of healthy children.
